# Developmental Plasticity in *Protea* as an Evolutionary Response to Environmental Clines in the Cape Floristic Region

**DOI:** 10.1371/journal.pone.0052035

**Published:** 2012-12-13

**Authors:** Jane E. Carlson, Kent E. Holsinger

**Affiliations:** Department of Ecology and Evolutionary Biology, University of Connecticut, Storrs, Connecticut, United States of America; University of Kent, United Kingdom

## Abstract

Local adaptation along steep environmental gradients likely contributes to plant diversity in the Cape Region of South Africa, yet existing analyses of trait divergence are limited to static measurements of functional traits rather than trajectories of individual development. We explore whether five taxa of evergreen shrubs (*Protea* section *Exsertae*) differ in their developmental trajectories and capacity for plasticity using two environmentally-distinct common gardens in South Africa. We measured seedlings in the summer-dry season and winter-wet season of each of two consecutive years to characterize ontogeny and plasticity within years, as same-age leaf cohorts mature, and between years, i.e., from leaf one cohort to the next. We compared patterns of development between gardens to assess whether trait trajectories are programmed versus plastic and examined whether developmental differences covaried with characteristics of a seedling’s home environment. We detected plasticity in developmental trajectories for leaf area, stomatal size, stomatal pore index, and to a limited extent specific leaf area, but not for stomatal density. We showed that the species growing in the harshest environments exhibits both the smallest increase in leaf area between years and the least change in SLA and photosynthetic rates as leaves age within years. These results show that within this clade, species have diverged in developmental trajectories and plasticity as well as in mean trait values. Some of these differences may be associated with adaptation to cold and drought stress within an environmentally-complex region.

## Introduction

Environmentally heterogeneous regions often have higher plant species diversity than would be expected from their size or latitude [1], an observation that has generated wide-spread interest in how this diversity evolved and is maintained [2]–[6]. Explanations include increased ecological space for co-existence [1], increased non-adaptive differentiation associated with complex biogeographic histories [2], [7]–[9], and increased opportunity for adaptive divergence and ecological speciation [10], [11]. A role for local adaptation in the generation of diversity is supported by the many studies showing that strong environmental gradients promote divergence in plant functional traits –i.e., those that affect local survival and performance– among populations and closely-related species [12]–[19]. While these and related studies focus on traits at one point in time, trait values change as plants grow and the trajectories followed during development may also diverge due to selection. The potential for this class of genetically-based differences among populations and species is largely unexplored.

Individual plants may differ in how their traits change during development either as a result of genetic differences (fixed ontogeny), environmental differences that induce phenotypic change (plasticity), or some combination of both. Although the relative contributions of genetic differentiation and plastic responses to local adaptation are not well understood [20]–[22], there are good reasons to expect that trajectories will be relatively fixed in harsh environments and more environmentally responsive, i.e. plastic, in those that are benign and highly variable. Environments where water and nutrients are relatively scarce, for example, may favor plants with minimal change along canalized trajectories (i.e. fixed ontogenies) because the benefit of conserving resources are great and the costs of producing the ‘wrong’ phenotype are high [23], [24]. Similarly, environments that are highly heterogeneous over time may favor plants with environmentally-responsive trajectories because closer trait-environment matching should improve plant performance. Such evolved responsiveness is particularly likely in sites where environmental change is at least partially unpredictable and occurs at a time-scale to which plants can detect and respond [21], [25]–[28].

The Cape Floristic Region (CFR) of South Africa is characterized by exceptional regional plant diversity –9,000 plant species in 90,000 km^2^ [7]– and extreme environmental heterogeneity. Not only is there great spatial heterogeneity in the total amount of rainfall and mean annual temperature, for example, but there are also sharp differences among sites in the degree of seasonality. The gradient in rainfall seasonality is particularly striking. In less than 500 km from east to west, the timing and intensity of peak rainfall varies from aseasonal to over half of annual rain falling within three winter months. Previous work on white proteas (*Protea* section *Exsertae*; Proteaceae) shows that gradients in rainfall seasonality, summer drought severity, and winter temperature contribute to among-population adaptive differentiation in several leaf traits measured from six-month old plants [18], but differences in the trajectories these traits follow as plants grow have yet to be explored.

**Figure 1:**
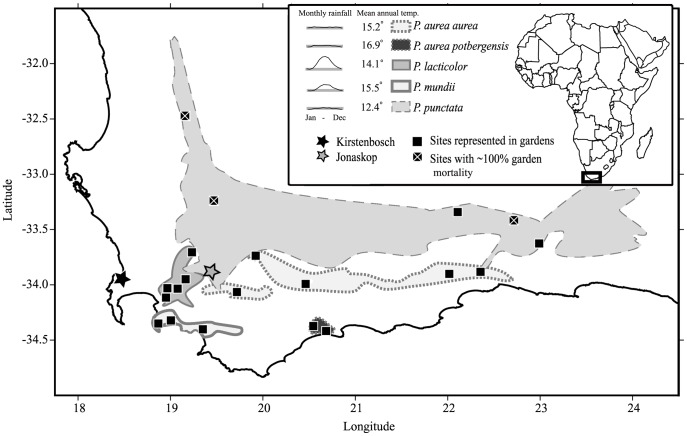
Geographic ranges and sampling sites of the five white protea taxa (*Protea* section *Exsertae*) used in this study. The locations of the Kirstenbosch and Jonaskop common gardens are starred on the map. The ranges for each species encompass all populations in the Protea Altas Database [72], except for *P. mundii*, which is represented by only the western half of its disjunct distribution (see also Materials and Methods). Median monthly rainfall trendlines and mean annual temperature values are based on 30+ year climate means from Schulze [49] intersected with the Protea Atlas datapoints used for range areas. The grey bars underlying the rainfall trendlines serve as a baseline for comparison, spanning from 15 to 45 mm rainfall. The species-wide climate means shown here are broadly similar to means based only on sampled populations, except for *P. punctata* which is slightly colder and drier in the species-wide average.

**Figure 2:**
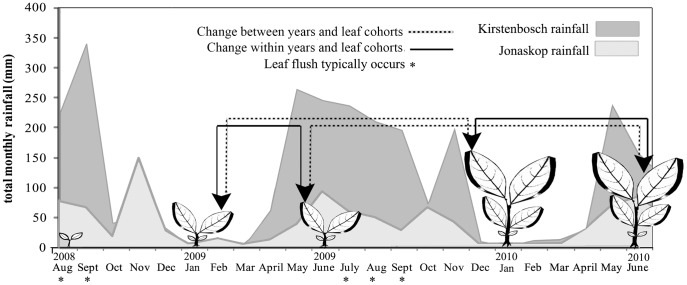
Sampling scheme for five white protea taxa in the Kirstenbosch and Jonaskop common gardens in South Africa. Solid lines track leaves from the same cohort within years, representing individual leaf ontogeny. Dotted lines track leaves from consecutive annual cohorts, representing whole seedling ontogeny. Rainfall data was measured on-site for Kirstenbosch and within 5 km of the garden for Jonaskop.

Here we examine the contribution of plasticity and fixed developmental trajectories to among-population and among-species differences in *Protea* section *Exsertae*. Specifically, we focus on five taxa from Carlson et al. [18] that differ broadly in mean climates and intra-annual variation in rainfall (Fig. 1). Among these species, *Protea lacticolor* experiences the most seasonally skewed rainfall, and *Protea punctata* experiences the coldest temperatures and least rainfall on average. We use seedlings from 17 populations to characterize the developmental trajectories of leaf traits over two years in two environmentally-distinct common gardens. We focus on seven leaf traits likely to affect plant performance and survival under different environmental conditions [29]–[31], many of which were also included in Carlson et al. [18]. By measuring these traits on seedlings in the summer (dry) and winter (wet) of each of two consecutive years, we characterize trait differences both *within* and *between* years (Fig. 2). Because leaf flush occurs in the austral spring of each year, our *within-year* measures are on leaves from the same annual cohort, representing the ontogeny of individual leaves. Our *between-year* measures are on leaves from consecutive annual cohorts, representing the ontogeny of whole seedlings.

By measuring *Protea* seedlings in outdoor gardens, we observe seedling development under changing environments as occur in the wild. However, these seasonal and annual fluctuations also confound our measures of ontogeny within each common garden. We therefore focus our measures of plasticity on the differences between plants grown in contrasting environments from the same maternal family, rather than focusing on within-individual responses over time. By comparing trait trajectories between gardens, we can assess whether trait trajectories are relatively fixed (gardens similar) or plastic (gardens differ). We use these and other comparisons to address the following questions:

1. How do traits change within and between annual leaf cohorts, and how do traits co-vary?
2. Which leaf traits are plastic and which reflect fixed trajectories?
3. Do species differ in how much leaves change *between annual leaf cohorts*, and are differences associated with home-site measures of resource availability?
4. Do species differ in how much leaves change from *dry to wet season within leaf cohorts*, and are differences associated with home-site measures of resource availability or rainfall seasonality?

## Materials and Methods

### Ethics Statement

Seeds were collected under permits from Cape Nature (AAA005–00093–0028 and AAA005–00125–0028), Ezemvelo KZN (1789/2008), Eastern Cape Parks Board and Department of Water Affairs and Forestry (CRO.23/08CR). *Protea lacticolor* is listed as “endangered” on the Red List of South African Plants because of the small number of known populations and its restricted geographical distribution (http://redlist.sanbi.org/species.php?species=799-63). Other taxa included in this study are listed as “least concern”.

### Study Species

We studied leaf-trait differences between and within annual leaf cohorts in five members of the white protea clade endemic to the Western Cape of South Africa: *P. aurea* (Burm. f.) Rourke ssp. *aurea*, *P. aurea* ssp. *potbergensis* (Rourke) Rourke, *P. lacticolor* Salisb., *P. mundii* Klotzsch, and *P. punctata* Meisn. The remaining two members of the white protea clade, *P. venusta* and *P. subvestita*, were included in the original experimental design, but their first-year seedling mortality was nearly 100% (similarly for western populations of *P. punctata*; Fig. 1). Moreover, *P. subvestita* does not occur in the CFR, representing an evolutionary “escape” to the Eastern Cape, Kwa-Zulu Natal, and Lesotho [32], and *P. venusta* is a low-growing, prostrate, mat-forming species. Recent microsatellite work shows that western *P. mundii* populations are genetically distinct from the geographically disjunct eastern populations of the species [33]; and only western populations were used in this study.

Monophyly of the white proteas is well-supported within *Protea* [9], [32], and members of the clade diverged from a common ancestor 0.34–1.2 MYa [33]. All studied species are evergreen, broad-leaved, sclerophyllous shrubs growing to 4 m or more in height. They take 2–3 years to reach reproductive maturity, after which they flower annually and store seeds aboveground in serotinous infructescences. Seeds are released when adult plants are killed by fire, which occurs at 15–40 year intervals [34]. [33]. On average, populations of *P. punctata* followed by *P. aurea* subsp. *potbergensis* receive the least rainfall, and those of *P. punctata* followed by *P. lacticolor* experience the coldest temperatures (Fig. 1).

### Sampling Design and Common Gardens

We collected seeds from the 17 study populations between February and April 2008, as part of the larger sampling effort described in Carlson et al. [18]. Our study included five populations of *P. aurea* subsp. *aurea*, two of the narrowly distributed *P. aurea* subsp. *potbergensis*, five of *P. lacticolor*, three of *P. mundii*, and two of *P. punctata* (Fig. 1). In each study population, we collected 5 infructescences from each of 13–20 adult plants (mean n = 17) and dried them at low humidity until achenes were released. We selected for germination only the plump seed-filled achenes that were undamaged by seed-eating larvae [35].

We germinated seeds in a greenhouse at Kirstenbosch in May 2008 and transplanted up to 21 seedlings per population (305–325 total; seedlings <2 cm in height, just prior to first flush of juvenile leaves) into each of two gardens in July 2008 (see Fig. 1. for garden locations; see also Carlson et al. [18]). The two gardens span the natural range of climate variability for studied white proteas (Carlson et al. 2011). The warmer, moister garden at Kirstenbosch Botanical Garden (175 m elevation), received 1745 and 1398 mm rainfall in the first and second years of study, respectively (from July 1 each year; L. Nurrish pers. comm.; Fig. 2). The colder, drier garden on Jonaskop mountain (944 m elevation) received 612 mm rainfall in the first year and 429 mm in the second (G. Midgley pers. comm.; Fig. 2). Soil fertility is higher at Kirstenbosch than at Jonaskop (Kirstenbosch: total P 12 ppm, total K 171 ppm, total N 0.86%; Jonaskop: total P 7 ppm, total K 126 ppm, total N 0.75%). We planted each garden with two or more offspring from the same 109 maternal lines (≤7 lines per population), but by the first measurement, 37–41% of seedlings had died in each garden.

At six-month intervals starting in January 2009, we measured leaf traits of all seedlings (Fig. 2). We timed measurements to capture trait values in the mid-dry season/summer (January) and mid-wet season/winter (June) of the first and second year post-germination. Leaf flush typically occurred just after the wet-season measure in June, so the second year of study focused on a new annual cohort of leaves. At each measurement interval, we collected from each live seedling a fully expanded leaf at or near the plant apex. We measured stomatal size and stomatal density under a light microscope, using cellophane tape peels (following [36]) taken from the abaxial leaf surface only. Previous studies on seven *Protea* species showed equal distribution of stomata on both leaf sides [37], [38]. We calculated stomatal pore index as guard cell length^2^ × stomatal density [39]. We also measured fresh leaf area using a LiCor 3100 leaf area meter (Lincoln, NE). We then dried the leaves for two weeks at 60°C, weighed them, and calculated SLA (leaf area divided by dry leaf mass; cm^2^/g). In the Kirstenbosch garden, we also measured light-saturated photosynthetic rates per unit leaf area (*A*_a_; µmol CO_2_×m^−2^×s^−1^) and stomatal conductance (*g*; mol H_2_O×m^−2^×s^−1^) between 0800 and 1000 h on clear days within 5 weeks from the start of each interval. We measured fully-expanded leaves, still attached to the plant, using a LiCor 6400XT with CO_2_ mixing system and a red/blue LED light source (Lincoln, NE; CO_2_ concentration = 400 µmol×m^−1^, PAR = 1500 µmol×m^−2^×s^−1^ and mean relative humidity = 39–44%, additional details in Carlson et al. [18]). Measurements were taken at cooler temperatures in the two wet seasons (21 and 26°C, respectively) and warmer temperatures in the two dry seasons (29 and 30°C). We also calculated photosynthesis per unit leaf mass (*A*_m_ µmol CO_2_×g^−1^×s^−1^), but given its strong correlation with *A*_a_, we included it only in analyses of inter-trait correlations.

### Statistical Analyses

#### Q1: How do seedling traits change within and between annual leaf cohorts, and how do traits co-vary?

We examined the trajectories of trait change in *Protea* seedlings from the dry to wet seasons within annual leaf cohorts as well as from one year to the next on consecutive leaf cohorts. We classified each of the four 6-month time steps by season (dry/January or wet/June) and by year (2009 or 2010) and determined whether leaf traits differed significantly between seasons (within leaf cohorts), years (between leaf cohorts), among species or involved significant 2 or 3-way interactions using linear mixed models in Proc MIXED (SAS 9.2; Cary, NC). Each trait was analyzed in a separate model, and we accounted for correlations among measurements on the same seedling using the repeated statement and an unstructured covariance structure (type = UN or ARH(1), chosen based on AIC model comparisons; Moser 2004). Additional random effects were population nested in species and its two and three way interactions. Because sampling was unbalanced among species, we estimated denominator degrees of freedom using the Kenward-Roger approximation [40]. We log-transformed leaf area, stomatal density, and stomatal pore index to improve normality of residuals. To ensure that our results did not reflect changes in trait values due to differential mortality among seedlings within gardens, we included only the seedlings that survived to the end of the study (n = 61 seedlings in Kirstenbosch and n = 152 in Jonaskop). Because of the small sample sizes remaining in the Kirstenbosch garden, our ability to detect between-garden differences in trait trajectories is limited. Nonetheless, we analyzed each garden separately because a preliminary analysis revealed significant interactions with garden for every trait. Moreover, we compared trait trajectories between gardens using only the subset of maternal lines that were shared between gardens to ensure that differences in the genetic composition of each garden (due to mortality) did not confound our assessment of environmental responsiveness.

We were unable to measure *A*_a_ and *g* on 27% of focal individuals in June 2009. Rather than exclude those individuals, we imputed missing values with proc MI, which employs multiple regression techniques to estimate missing values [41], [42]. Our imputations used all prior and subsequent trait measurements of those seedlings, as well as their species and population designation. We ran five sets of imputations, analyzed each dataset separately using the above model in Proc MIXED, and combined results using proc MIANALYZE (SAS 9.2, Cary, NC; [42]–[44]). The signs of regression coefficients and the results of significance tests using imputed data were equivalent to those from similar analyses on all individuals in 2010 when there were no missing values.

To assess correlations between and within leaf morphological and physiological traits, we estimated pair-wise correlations, analyzing each of the four time-steps in each garden separately. Within each time-step, we included all individuals surviving to that time step. All leaf traits were compared, as well as the additional variable of photosynthesis per unit leaf mass (*A*_m_). Because of the large number of pairwise tests, we used Bonferroni-adjusted alpha levels in tests of significance.

#### Q2: Which leaf traits are plastic and which reflect fixed trajectories?

To determine which trait trajectories for leaf and stomatal traits were plastic, we compared trajectories for maternal lines that survived to the final time step of within- or between annual leaf cohort comparisons. Because the number of maternal lines decreased with time, we used only 2009 data for maternal-line trait comparisons within years and cohorts (n = 47) and January to January differences for maternal-line trait comparisons between years and cohorts (n = 39). We performed an additional analysis on SLA comparing its initial to final measurement between gardens, because this trait changed between both season and years (n = 31). All species and populations were represented in each of these analyses. We averaged trait values for seedlings from the same maternal line within gardens (<4 seedlings) and calculated the mean difference in trait means (▵) within and between years for each garden. We used the seasonal or annual change in each trait value in each garden as the response variables in repeated measures models (repeated subject = maternal line) in Proc MIXED. The only fixed effect was garden, and random effects were species and population nested in species. Given that *A*_a_ and *g* were for Kirstenbosch only, we could not determine whether trajectories in these traits were plastic.

#### Q3: Do species differ in how much leaves change *between annual leaf cohorts*, and are differences associated with home-site measures of resource availability?

We determined whether species differed in how much traits changed between annual leaf cohorts as part of the analyses of trait trajectories on each garden separately (Question 1). If the full mixed model had a significant interaction between year and species, we determined which species differed by comparing 95% confidence intervals around differences in LS-Means (t-type intervals; SAS Institute Inc. [45]). As a conservative test, we regarded species as having significantly different degrees of change only if the confidence intervals did not overlap. When there were three way interactions, we compared 95% confidence intervals around differences in LS-Means separately for January and June between-year intervals and 2009 and 2010 within-year intervals. Because we *a priori* expected at least some species to differ for some traits and we only compared differences after finding significant species interactions, we did not make further adjustments to control the type I error rate (see also [46], [47]).

To supplement species level comparisons, we used multiple regressions to determine whether the degree of change between annual leaf cohorts is related to environmental characteristics of source populations. Within-garden changes in SLA, *A*_a_, *g*, leaf area, pore index, stomatal size, and stomatal density were regressed separately against three measures of home-site resource availability. Species and population nested in species were random effects in all models. To maximize sample sizes, we compared trait change only between Jan. 2009 and Jan. 2010 (Kirstenbosch = 72, Jonaskop = 173).

We represented the seedling’s environmental source using the first axis of each of three principal component analyses, one related to dry season drought, one related to winter temperatures, and the third related to soil fertility (same as in Carlson et al. [18] and Prunier et al. [48]). COLDPCA is based on the mean daily minimum temperature in the coldest month (July) and the total heat units over 10°C for the three coldest winter months. DRYPCA represents the number of days without rain during the driest three months and the total amount of rain to fall within those months. FERTPCA represents the amount of N (%), total P (mg/kg), and total K (mg/kg) in sub-surface (up 30 cm) soil samples averaged across three locations at each site (see Carlson et al. [18] for additional details). High values of DRYPCA, COLDPCA, and FERTPCA represent relatively mild drought, warm winters, and more fertile soils, respectively. Climate data were derived from the South Africa Atlas of Climatology and Agrohydrology [49]. The soil samples used for soil fertility analyses were collected at the same time as the seeds for the gardens, and they were analyzed for extractable macro nutrients at BEM labs, South Africa (see Carlson et al. [18]).

#### Q4: Do species differ in how much leaves change from *dry to wet season within leaf cohorts*, and are differences associated with home-site measures of resource availability or rainfall seasonality?

We used methods similar to those we used for Question 3 to determine whether species or populations differed in the degree of change from dry to wet seasons within annual leaf cohorts. For species-level analyses, we used results from the model in Question 1 to identify traits that showed a significant interaction between species and season, indicating that trajectories differed among species. For trajectories showing among species differences, we tested the significance of species-level differences using the 95% confidence intervals around differences in LS-Means. For population-level analyses, we considered only traits that differed significantly between seasons within years in the species-level analyses, and we compared change only between dry and wet seasons in 2009 (Kirstenbosch n = 91; Jonaskop n = 177). To analyze population-level data, we used the same multiple regression approach as in Question 3 except that we added an environmental variable representing rainfall seasonality, PPTCON. PPTCON is the proportion of total annual precipitation to fall in a single month [49], and low values are associated with aseasonal rainfall throughout the year. For *A*_a_ and *g* in population-level analyses, we performed the same multiple regressions, but we used the imputed data for June 2009. Results for the five analyses including imputed data were again combined in MIANALYZE (SAS 9.2).

## Results

### Q1: How do Seedling Traits Change within and between Annual Leaf Cohorts, and how do Traits Co-vary?

**Figure 3:**
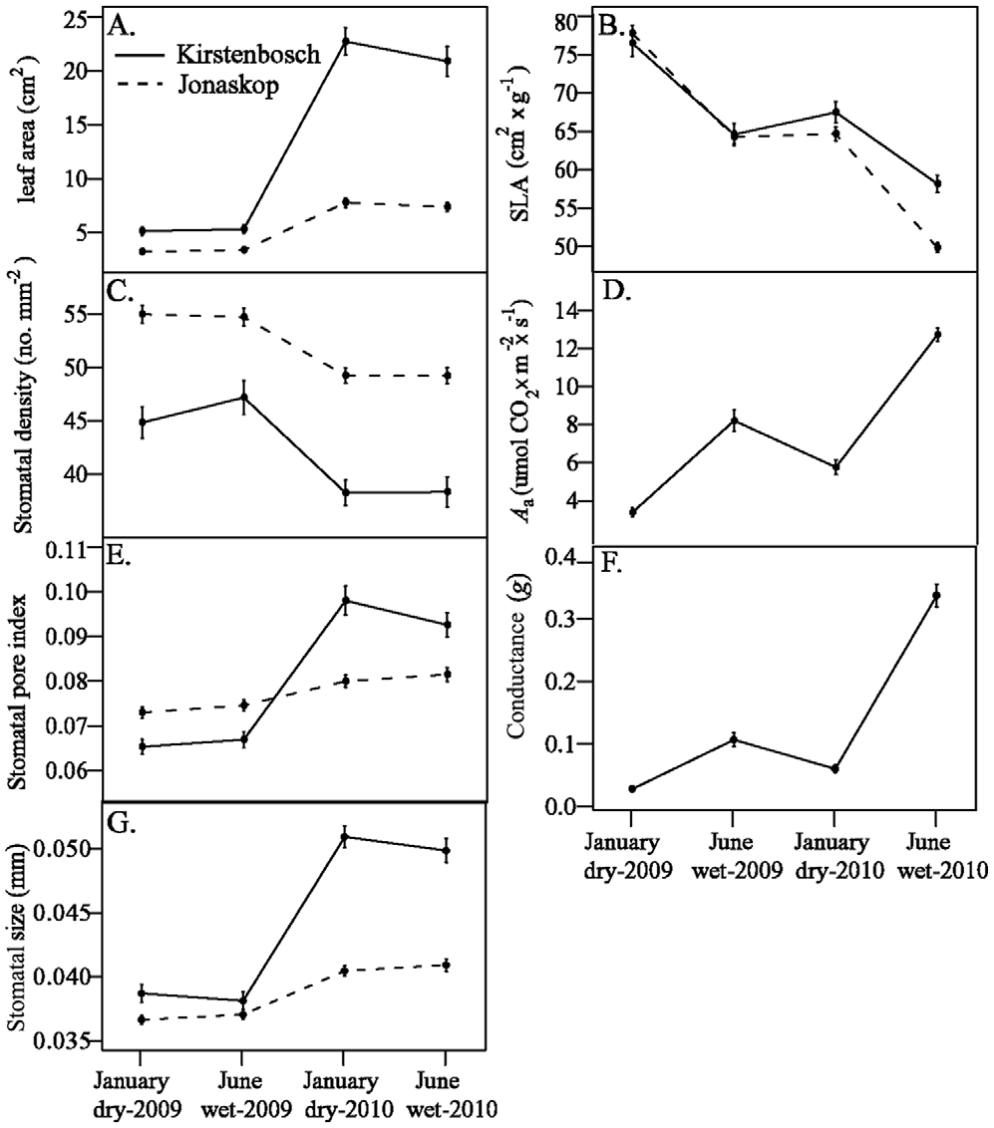
Change in (A) leaf area, (B) SLA, (C) stomatal density, (D) light-saturated photosynthetic rate, *A*_a_, (E) stomatal pore index, (F) stomatal conductance, *g*, and (G) stomatal size of seedlings of five white protea taxa between the dry (January) and wet (June) seasons of 2009 and 2010 in common gardens at Kirstenbosch and Jonaskop in the Western Cape, South Africa. Traits *A*_a_ and *g* were measured only in the Kirstenbosch garden, whereas the remaining traits were measured in both gardens. Only seedlings that survived till the end of the study (June 2010) were used to calculate the raw means and error bars (±1 SE) shown here.

Within each garden, all measured *Protea* leaf traits differed significantly between annual leaf cohorts (significant year effect), but only SLA and *A*_a_ also changed within cohorts (significant season effect: Fig. 3, Table 1). Second-year leaves were larger than first-year leaves (Fig. 3A), and they had lower SLA (Fig. 3B), fewer stomata per mm^2^ (Fig. 3C), higher light-saturated photosynthetic rates (Fig. 3D), more leaf surface dedicated to gas transport with higher conductance (Fig. 3E-F), and larger stomata (Fig. 3G). As leaves matured between the dry (Jan.) and wet (June) season within each year, SLA decreased and *A*_a_ increased (Fig. 3B, D). In Kirstenbosch, there was also a significant interaction between year and season for leaf area, corresponding to a slight but significant decrease between seasons in 2010 (Tukey-adjusted *p* = 0.05), but not in 2009 (*p* = 0.22). Although patterns of trait change were similar between gardens, between-year changes were greater for the moister Kirstenbosch than the drier Jonaskop garden for all traits except SLA (Fig. 3).

**Table 1:** Results of linear mixed models testing whether leaf traits of white protea seedlings differ based on the year of measurement, the season of measurement, species effects, or their interaction.

				SLA (cm^2^ × g^−1^)			Leaf area (cm^2^)			Stomatal density(no. × mm^−1^)			Stomatal pore index (density × length^2^)			Stomatal size(length in mm)			*A*_a_(µmol CO_2_ × m^−2^× s^−1^ )			Stomatal conductance, *g* (mol H_2_O×m^−2^ ×s^−1^)		
Garden	Predictors		*F*		*p*-value	*F*		*p*-value	*F*		*p*-value	*F*		*p*-value	*F*		*p*-value	*F*		*p*-value	*F*		*p*-value	
Kirstenbosch	year		30.1		**<0.0001**	256.1		**<0.0001**	17.4		**0.002**	153.5		**<0.0001**	259.3		**<0.0001**	8.7*		**0.0005**	−3.9*		**<0.0001**	
	season		122.5		**<0.0001**	3.5		0.07	1.1		0.31	0.02		0.90	1.1		0.31	51.6*		**<0.0001**	−7.9*		**<0.0001**	
	year*season		0.4		0.54	5.1		**0.03**	0.1		0.71	0.6		0.46	0.2		0.66	6.5*		0.07	−1.7*		0.09	
	species		14.5		**<0.0001**	15.7		**<0.0001**	4.8		**0.01**	0.7		0.59	5.0		**0.017**	3.5*		0.06	2.3*		0.10	
	year*species		0.9		0.49	7.8		**<0.0001**	3.1		0.08	2.8		**0.04**	1.7		0.17	0.03*		0.50	−0.4*		0.78	
	season*species		3.6		**0.03**	1.4		0.25	2.6		0.09	1.5		0.27	0.6		0.68	7.6*		**0.006**	−1.7*		0.24	
	year*season*species		0.81		0.53	1.5		0.21	0.7		0.57	1.2		0.32	0.5		0.74	1.82*		0.18	−1.9*		0.20	
Jonaskop	year		208.9		**<0.0001**	148.8		**<0.0001**	38.6		**<0.0001**	27.2		**<0.0001**	59.8		**<0.0001**	**-**		**-**	**-**		**-**	
	season		349.5		**<0.0001**	1.4		0.23	0.2		0.69	0.8		0.39	3.4		0.065	-		-	-		-	
	year*season		7.5		**0.007**	5.3		**0.02**	0.01		0.91	0.03		0.86	0.01		0.93	-		-	-		-	
	species		14.1		**0.0001**	20.2		**<0.0001**	6.4		0.004	4.3		0.02	7.2		0.0028	**-**		**-**	**-**		**-**	
	year*species		6.9		**0.003**	6.7		**<0.0001**	1.7		0.22	1.1		0.36	0.4		0.81	-		-	-		-	
	season*species		1.6		0.19	0.2		0.93	0.2		0.94	0.5		0.77	0.6		0.68	-		-	-		-	
	year*season*species		4.3		**0.003**	1.1		0.38	0.3		0.85	0.4		0.83	0.09		0.98	-		-	-		-	

The two gardens were analyzed separately using Proc MIXED with repeated measures on seedlings and an unstructured (UN) covariance structure. Log transformations were performed as needed to improve the normality of residuals. n = 52 plants for Kirstenbosch (except SLA with 51) and 161 for Jonaskop. Sixteen missing values for *A*_a_ and *g* were imputed with proc MI and MIANALYZE in SAS 9.3. Log transformations were used on stomatal density, pore index, and conductance. Asterisk indicates theta values from MIANALYZE rather than F-values.

A few leaf traits were strongly inter-correlated within gardens and time steps, based on Pearson’s correlation coefficients (r; Table 2). The strongest correlations were between *g* and *A*_a_ or *A*_m_ (r = 0.70 to 0.92), *A*_a_ and *A*_m_ (r = 0.82 to 0.96), stomatal size and stomatal density (r = −0.45 to −0.74), stomata size and pore index (r = 0.36 to 0.77), stomatal size and leaf area (r = 0.55 to 0.65), pore index and leaf area (r = 0.31 to 0.42), and stomatal density and leaf area (r = −0.21 to −0.58). All correlations between other morphological traits and conductance or photosynthesis were weak and non-significant, with the highest involving leaf area in January 2009, at −0.26 for *g* and −0.20 for *A*_a_ (Table 2).

**Table 2:** Pair-wise correlations of all measured traits, compared separately for each time of measurement and each garden.

Garden	Trait	Trait	January 2009	June 2009	January 2010	June 2010
Kirstenbosch	Pore index	Stomatal density	0.1700.10	0.1410.17	**0.421** **0.0001**	0.3550.009
	Pore index	SLA	−0.1600.11	−0.2280.03	0.00150.99	0.1090.44
	Pore index	Stomatal size	**0.540** **<0.0001**	**0.609** **<0.0001**	**0.567** **<0.0001**	0.3960.003
	Pore index	Leaf area	**0.379** **0.0002**	**0.417** **<0.0001**	**0.371** **0.0009**	−0.00430.98
	Pore index	*A*_a_	0.0800.44	0.1250.31	0.02090.86	0.0480.74
	Pore index	*A*_m_	0.03410.75	0.1200.33	0.06360.59	0.1030.47
	Pore index	*g*	0.1090.30	0.03190.80	0.07400.53	0.1190.41
	Stomatal density	SLA	0.1980.001	0.02310.82	0.2660.02	0.1950.16
	Stomatal density	Stomatal size	−**0.717** **<0.0001**	−**0.682** **<0.0001**	−**0.488** **<0.0001**	−**0.692** **<0.0001**
	Stomatal density	Leaf area	−**0.409** **<0.0001**	−**0.331** **0.001**	−0.2180.06	−**0.578** **<0.0001**
	Stomatal density	*A*_a_	0.08130.33	0.00850.95	−0.09750.41	−0.03530.81
	Stomatal density	*A*_m_	0.09710.25	−0.01710.89	0.03170.79	0.05770.69
	Stomatal density	*g*	0.1590.06	−0.09640.44	−0.03160.79	0.1710.23
	SLA	Stomatal size	−0.2730.007	−0.1990.05	−0.2430.04	−0.1390.32
	SLA	Leaf area	−**0.255** **<0.0001**	−0.2710.008	−0.2310.05	−0.2660.05
	SLA	*A*_a_	−0.09290.27	−0.1660.18	−0.2070.08	0.040.77
	SLA	*A*_m_	0.2450.003	0.06660.59	0.1020.391	**0.595** **<0.0001**
	SLA	*g*	−0.09690.25	−0.1000.43	−0.1940.10	0.00470.97
	Stomatal size	Leaf area	**0.591** **<0.0001**	**0.599** **<0.0001**	**0.532** **<0.0001**	**0.557** **<0.0001**
	Stomatal size	*A*_a_	0.1480.16	0.08080.52	0.1170.32	0.007270.96
	Stomatal size	*A*_m_	0.08380.42	0.09510.44	0.03100.79	−0.05190.712
	Stomatal size	*g*	0.1170.26	0.09220.47	0.1010.39	−0.1430.32
Garden	Trait	Trait	January 2009	June 2009	January 2010	June 2010
Kirstenbosch	Leaf area	*A*_a_	−0.2030.01	0.04290.73	0.001530.99	0.09110.53
	Leaf area	*A*_m_	−0.2450.003	0.001090.99	−0.02630.83	−0.07100.62
	Leaf area	*g*	−0.2610.002	0.03550.78	−0.003010.98	0.02540.86
	*A*_a_	*g*	**0.889** **<0.0001**	**0.840** **<0.0001**	**0.930** **<0.0001**	**0.700** **<0.0001**
	*A*_m_	*g*	**0.796** **<0.0001**	**0.824** **<0.0001**	**0.870** **<0.0001**	**0.569** **<0.0001**
	*A_a_*	*A*_m_	**0.921** **<0.0001**	**0.967** **<0.0001**	**0.945** **<0.0001**	**0.819** **<0.0001**
Jonaskop	Pore index	Stomatal density	**0.308** **<0.0001**	**0.300** **<0.0001**	0.1930.01	0.03980.62
	Pore index	SLA	−0.1800.02	−**0.228** **0.002**	−0.08430.27	−0.1160.15
	Pore index	Stomatal size	**0.624** **<0.0001**	**0.700** **<0.0001**	**0.677** **<0.0001**	**0.777** **<0.0001**
	Pore index	Leaf area	**0.417** **<0.0001**	**0.306** **<0.0001**	**0.394** **<0.0001**	**0.381** **<0.0001**
	Stomatal density	SLA	0.03280.62	0.08350.27	0.04790.53	**0.259** **0.001**
	Stomatal density	Stomatal size	−**0.534** **<0.0001**	−**0.454** **<0.0001**	−**0.571** **<0.0001**	−**0.572** **<0.0001**
	Stomatal density	Leaf area	−**0.292** **<0.0001**	−**0.335** **<0.0001**	−**0.393** **<0.0001**	−**0.470** **<0.0001**
	SLA	Stomatal size	−0.1230.10	−**0.261** **0.0004**	−0.1260.10	−**0.245** **0.002**
	SLA	Leaf area	−**0.222** **0.0007**	−**0.344** **<0.0001**	−0.09430.22	−**0.289** **0.0003**
	Stomatal size	Leaf area	**0.654** **<0.0001**	**0.554** **<0.0001**	**0.648** **<0.0001**	**0.622** **<0.0001**

The upper value is the Pearson’s r and the lower value is the p-value for a simple linear regression. Measures of light-saturated photosynthetic rate are indicated by *A*_a_ area-based measures and *A*_m_ for mass-based measures. Stomatal conductance is indicated by *g*. To correct the p-value for multiple tests within gardens and within time steps, the adjusted p-value for Jonaskop is 0.005 and for Kirstenbosch it is 0.0018. Statistically significant correlations are bolded.

### Q2: Which Leaf Traits are Plastic and which Reflect Fixed Trajectories?

Three of five traits were plastic between annual leaf cohorts, as evidenced by trajectory differences between gardens using only shared family lines. Leaf area, stomatal size, and stomatal pore index each changed more in Kirstenbosch than in Jonaskop (▵leaf area: F_1,60_ = 103.2, p<0.0001; ▵ stomatal size: F_1,60_ = 46.3, p<0.0001; ▵ pore index: F_ 1,60_ = 26.97, p<0.0001). In contrast, we failed to detect a significant garden effect for ▵SLA (F_1,60_ = 1.59, p = 0.21) or ▵stomatal density (F_1,60_ = 0.07, p = 0.79), suggesting that the trajectories for SLA and stomatal density differ little between gardens. We detected no significant differences between gardens for any traits in the degree of within-cohort change in 2009 (F_1,76_<1.03, p>0.32). For SLA alone, however, the combination of within- and between-cohort change resulted in a 2-year trajectory that differed significantly between gardens (▵SLA from Jan. 2009 to June 2010: F_1,44_ = 8.03, p = 0.007).

### Q3: Do Species Differ in How Much Leaves Change *between Annual Leaf Cohorts*, and are Differences Associated with Home-site Measures of Resource Availability?

**Figure 4:**
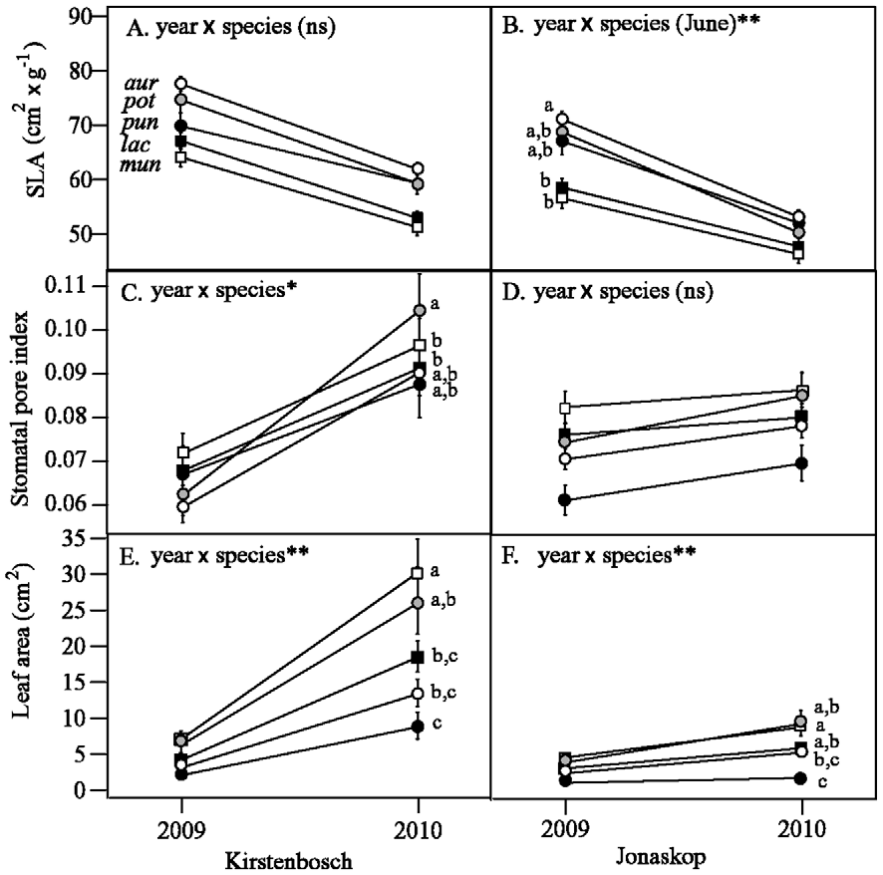
Between-year developmental trajectories of seedling leaf traits SLA (A,B), stomatal pore index (C,D), and leaf area (E,F) for white protea taxa grown in common gardens at Kirstenbosch (A,C,E) and Jonaskop (B,D,F) in the Western Cape, South Africa. Only traits for which developmental trajectories differed significantly among species are depicted (see Table 1). For (B), trajectories differed significantly among species for Jan. 2009 to Jan. 2010 as well as June to June measures, but because patterns were similar only the latter is shown. Although ∼300 seedlings were initially planted in each garden in July 2008, mortality reduced final sample sizes to 52 seedlings at Kirstenbosch and 161 seedlings at Jonaskop. Significant differences among species are indicated by lowercase letters based on 95% confidence intervals around differences in LS-means from repeated measures linear mixed models (see methods for details). Points are least squares means for each species (averaging Jan. and June measurements within years for all but B) and error bars are ±1 SE. * = p≤0.05, ** = p≤0.01.

We detected differences among species in developmental trajectories from the first to second leaf cohort for SLA, leaf area, and stomatal pore index (Table 3; Fig. 4). At Kirstenbosch, SLA trajectories did not differ among species (Fig. 4A), but at Jonaskop, SLA changed the most in the white protea species that is intermediate on both temperature and rainfall axes, *P. aurea* subsp. *aurea* (Fig. 4B). We observed the smallest amount of change in SLA at Jonaskop in two moist-environment species, *P. lacticolor* and *P. mundii* for the June to June interval (Fig. 4B), as well as the species from the overall harshest environments, *P. punctata,* for the Jan. to Jan. interval only (results not shown). At Kirstenbosch, stomatal pore index increased the most for *P. aurea* subsp. *potbergensis*, which is from relatively warm and dry climates, and the least for *P. lacticolor* and *P. mundii* (Fig. 4C), but we did not detect differences in the degree of change among species in Jonaskop (Fig. 4D). Finally, in both Kirstenbosch and Jonaskop, leaf area increased the most in *P. mundii* and the least in *P. punctata* (Fig. 4E–F).

**Table 3:** Results of multiple regressions to test the associations of environmental variables and developmental trajectories for leaves from successive cohorts compared between years and leaves from the same cohort compared within years.

Between year		Kirstenbosch			Jonaskop		
Leaf trait	Variable	Slope	F	*p*	Slope	F	*p*
▵Leaf area	DRYPCA	−0.01	0.09	0.77	0.0	0.0	0.98
(cm^2^; log+10	COLDPCA	0.06	1.82	0.18	0.05	1.91	0.17
Transformation)	FERTPCA	0.06	2.41	0.13	−**0.07**	**6.47**	**0.01**
▵SLA	DRYPCA	−2.02	1.87	0.18	−1.4	1.86	0.17
(cm^2^×g^−1^)	COLDPCA	0.07	0.0	0.97	**−4.4**	**6.08**	**0.01**
	FERTPCA	0.36	0.04	0.85	0.1	0.0	0.95
▵pore index	DRYPCA	0.0	1.68	0.20	0.0	0.38	0.54
(guard cell	COLDPCA	0.0	2.77	0.10	0.0	0.10	0.75
length^2^× SD)	FERTPCA	0.0	0.67	0.42	0.0	0.23	0.63
▵stomatal density	DRYPCA	−1.22	0.9	0.35	0.23	0.05	0.82
(no. per mm^2^)	COLDPCA	0.46	0.06	0.80	0.5	0.11	0.74
	FERTPCA	0.45	0.09	0.77	1.4	0.93	0.34
▵ stomatal size	DRYPCA	0.0	0.27	0.61	0.0	0.15	0.70
(length, mm)	COLDPCA	0.0	0.24	0.62	0.0	2.52	0.12
	FERTPCA	0.0	0.11	0.74	0.0	2.31	0.13
▵*A*_a_	DRYPCA	−0.02	0.0	0.95	–	–	–
(µmol CO_2_	COLDPCA	−0.23	0.2	0.66	–	–	–
× m^−2^ × s^−1^ )	FERTPCA	−0.08	0.04	0.84	–	–	–
▵ *g*	DRYPCA	−0.14	0.57	0.45	–	–	–
mol × m^−2^ × s^−1^	COLDPCA	0.07	0.07	0.79	–	–	–
	FERTPCA	−0.14	0.33	0.57	–	–	–
**Within year**							
▵SLA	DRYPCA	−2.71	**7.53**	**0.008**	−0.88	0.67	0.41
(cm^2^ × g^−1^)	COLDPCA	0.66	0.19	0.66	**−3.31**	**3.69**	**0.056**
	FERTPCA	−1.44	1.45	0.23	−0.01	0.0	0.99
	PPTCON	0.17	1.49	0.23	−0.03	0.07	0.79
▵*A*_a_	DRYPCA	0.20	0.46	0.64	–	–	–
(µmol CO_2_ × m^−2^	COLDPCA	−0.12	−0.18	0.86	–	–	–
× s^−1^)	FERTPCA	−0.21	−0.39	0.70	–	–	–
	PPTCON	−0.03	−0.95	0.34	–	–	–
▵ *g*	DRYPCA	0.0	−0.82	0.41			
(mol H_2_O × m^−2^	COLDPCA	0.0	0.53	0.60			
× s ^−1^)	FERTPCA	0.0	0.03	0.98			
	PPTCON	0.0	0.1	0.92	–	–	–

Bolded text denotes significant or marginally-significant relationships. Denominator degrees of freedom were 156 or 160 for Jonaskop and 54 or 73 for Kirstenbosch. For *A*_a_ and *g*, missing data for 17 seedlings in June 2009 were imputed and analyzed in Proc MIANALYZE (see methods for details).

Multiple regression analysis revealed that the amount of change in SLA between annual leaf cohorts at Jonaskop was significantly associated with the intensity of winter cold in the source population (Table 3). Plants from warmer sites decreased SLA more between leaf cohorts than did those from colder sites. Changes in leaf area at Jonaskop were also associated with FERTPCA. Surprisingly, leaf area increased more between leaf cohorts in plants sourced from lower fertility environments (Table 3).

### Q4: Do Species Differ in How Much Leaves Change from *dry to wet season within leaf cohorts*, and are Differences Associated with Home-site Measures of Resource Availability or Rainfall Seasonality?

**Figure 5:**
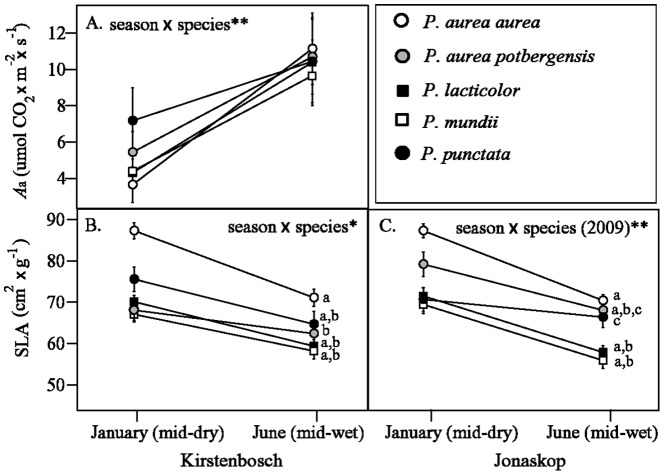
Within-year change in (A) light-saturated photosynthesis and (B,C) SLA of leaves measured in the dry and wet seasons year for white protea seedlings in Kirstenbosch (A,B) and Jonaskop (C) gardens in the Western Cape, South Africa. Only within-year trajectories that differed significantly among species are depicted (see **Table 1**). Significant differences among species are indicated by lowercase letters based on 95% confidence intervals around differences in LS-means from repeated measures linear mixed models (see methods for details). Points are least squares means for each species averaging 2009 and 2010 measurements within years for all but (C), and error bars are ±1 SE. * = ≤0.05, ** = p≤0.01.

In Kirstenbosch, the amount that *A*_a_ increased and SLA decreased from dry to wet season within leaf cohorts differed significantly among species (Fig. 5A). *Protea aurea* subsp. *aurea* increased *A*_a_ more within cohorts than did *P. punctata*, although this difference was not statistically significant. For SLA, *P. punctata* decreased the least between dry and wet seasons in Jonaskop, and *P. aurea* subsp. *potbergensis* decreased the least in Kirstenbosch, both differing significantly from *P. aurea* subsp. *aurea* (lowercase letters in Fig. 5B and 5C).

Multiple regression analysis showed that two home-site environmental variables were associated with the degree of change in SLA between wet and dry seasons within annual leaf cohorts (Table 3). At Kirstenbosch, plants increased their mass per unit leaf area (i.e., decreased SLA) more between seasons if sourced from wetter sites (high values of DRYPCA). At Jonaskop, SLA decreased more in plants derived from warmer sites (high values of COLDPCA), although this relationship was only marginally significant (Table 3).

## Discussion

Our results show that divergence in trait trajectories and their plastic responses are a relevant yet neglected aspect of the Cape Region of South Africa’s enormous diversity in plant form and physiology [2], [50], [51]. Although plant adaptive differentiation has long been of research interest (e.g., [12]), our study is one of few to investigate divergence among species in the trajectories that traits follow during plant development. Our earlier work showed that white protea populations differed in their specific leaf area, leaf area, and growth rate along environmental gradients in ways that appear to be adaptive [18]. Here we show that they also differ in their trait developmental trajectories and that some of these trajectories are plastic, responding to the environments in which the plants are grown. We also demonstrate that populations and species associated with drier or colder home climates show less change in some leaf traits between seasons and years in a common garden environment. Such associations between trait trajectories and environmental covariates suggest that in some cases, differences in both fixed developmental trajectories and plasticity may reflect adaptation to contrasting environments in the Cape Floristic Region.

### Plasticity Versus Genetic Differentiation in Trait Trajectories

The between-garden differences we found in trait trajectories for stomatal pore index, stomatal size, and leaf area indicate that these trajectories are plastic. For all of these traits, changes were much greater in the benign Kirstenbosch garden than in the harsher Jonaskop garden. The greater changes observed in Kirstenbosch seedlings could enhance seedling performance in the relatively moist, resource-rich environment by, for example, providing a larger total pore area dedicated to transpiration and gas exchange that could increase photosynthetic rates and carbon fixation [52]. In contrast, the shallower trajectories of these traits in Jonaskop could reflect resource, particularly water, conservation. If plasticity in stomatal traits is indeed increasing resource uptake at Kirstenbosch, we may expect strong positive associations between key stomatal traits and photosynthetic rates, as have been found elsewhere [31], [52], [53]. Photosynthetic rates at Kirstenbosch were not strongly correlated with stomatal pore index or stomatal size at any time step in our study, however, suggesting that such functional linkages are not consistent across species (see also [39], [54]). The lack of photosynthetic data from Jonaskop further limits our ability to interpret the functional significance of observed between-garden differences.

Trajectories for stomatal density and to a limited extent, SLA, were relatively undifferentiated between gardens, suggesting their developmental trajectories were less responsive to the contrasting garden environments than were those of the other traits. Alternatively, we may have failed to detect small between-garden differences due to sample size limitations. If the lack of detectable difference reflects common trajectories for both gardens, it could be the result of greater costs of plasticity, stronger selection for fixed values, or genetic constraints [24]. Our earlier work also found SLA to be the least plastic of all studied traits at 6 months post-planting [18], although others have often found it to exhibit large amounts of plasticity [55], [56].

### Plasticity and Leaf Trait Trajectories

The trait trajectories plants follow are determined, in part, by their genotypes. Hence, populations and species may differ in the extent to which traits change over the time and in the extent to which trait trajectories are plastic [57]. Because our sample includes only a few maternal lines per population, we cannot directly assess whether there are heritable differences in trait trajectories among family lines within populations. We do, however, have evidence for among-population and among-species differences in the trajectories for SLA, leaf area, and stomatal pore index, which also followed significantly different trajectories in each gardens. Thus, plasticity in these trajectories appears to have a heritable component.

Genetic differences among species may reflect local adaptation, accidents of history, differing constraints and strategies, or most likely, some of each. For example, the two taxa with the greatest plasticity for change in leaf area –western *Protea mundii* and *P. aurea* subsp. *potbergensis*–produce the largest leaves in the wild that are also relatively short-lived on the plant [18] (Carlson and Holsinger, unpublished), yet differ importantly in other ways. Western *P. mundii* occurs in warm, relatively moist climates, and stomatal pore index changes little from one cohort of leaves to the next. *Protea aurea* subsp. *potbergensis*, in contrast, occurs in drier, hotter climates and has a high capacity to increase both leaf area and the total stomatal pore area, possibility reflecting a strategy to draw water from dry soil more efficiently [31]. Although these patterns are suggestive, it remains to be demonstrated that the differences between these taxa reflect adaptation to different environments. Our results are at least consistent with others showing that closely related species may differ in the extent of plasticity [58], [59].

### Between-cohort Trait Trajectories and Differences Among Species

Studies of “ontogenetic contingency” [60], [61] have emphasized that the development of individual plant organs may be influenced both by the whole plant’s developmental stage and by the environmental conditions under which the organs develop. Classic examples of heteroblasty include those associated with differences between juvenile and adult leaves [62], [63] and those associated with aquatic and airborne leaves in aquatic plants [64], [65]. The heteroblasty between annual leaf cohorts we report here is more subtle, but it matches expectations of how leaf form and function should change across consecutive leaf cohorts produced by an individual plant. Jones [66] suggested that in the early stages of plant development, SLA should decrease, leaf area should increase, and photosynthetic rates should increase between leaves produced at different times. Our findings not only support these predictions at the whole-plant level, i.e., from one year or leaf cohort to the next, but they also match these expectations for how SLA and photosynthesis should change in older versus younger leaves from the same cohort (see below). The trajectories for stomatal traits seen here are also consistent with the literature [54]. Franks et al. [54] showed that *Acacia* seedlings had smaller stomata at higher densities than did resprouting adults in a common environment, even though the resprouted leaves were juvenile in morphology.

Grime [23] among others, has suggested that stress should reduce the capacity for rapid change in fixed trajectories and disfavor plasticity. Consistent with this view, we found that *P. punctata*, which grows in the coldest and driest sites, shows the smallest increases in leaf area between years. For other traits, however, *P. punctata* is not consistently the least plastic or slowest to change. *Protea lacticolor* and *P. mundii*, which both occur in wetter climates, showed the least change in stomatal pore index from one cohort to the next. These two species, along with *P. punctata*, also showed the least change in SLA between annual leaf cohorts. It may be that traits requiring the greatest resource allocation, namely leaf area, may be more moderated in harsh environments than those that require less [23]. Alternatively, differences in the patterns of change may reflect integrated responses, with traits unable to respond to selection independently.

Differences in trait trajectories among populations also align only partially with expectations based on home-site resource availability. As we predicted, plants from colder sites decreased SLA less than did those from warmer sites. In contrast, plants from relatively infertile sites appear to increase leaf area more than those in more fertile sites. Although this paradoxical result is difficult to interpret, it aligns with findings from Carlson et al. [18] that plants from less fertile sites had higher growth rates and that growth rates and leaf area were correlated in both gardens.

### Within-cohort Trait Trajectories and Differences Among Species

Individual leaves of white proteas live 3–5 years, and it is not surprising that some of their characteristics change as they grow older, at least during their first year. Indeed, our results show that specific leaf area decreases and maximum photosynthetic rate increases as leaves grow older within years. Because leaf flush occurs in July and August, this developmental pattern results in a temporal trait-environment mismatch: leaves are thin and flexible in January, when temperatures are high and rain is very infrequent, and thicker and more sclerophyllous in June when temperatures are low and rain is more common. The patterns of change in photosynthesis and conductance, in contrast, reflect a tighter match with immediate environmental conditions, as might be expected. It is not surprising that stomatal conductance and photosynthetic rates are lower in the hot, dry conditions experienced in the austral summer in the Western Cape. Lower stomatal conductance and photosynthesis under drought conditions have been observed across a wide range of taxa [67], [68].

Across both species and populations, differences in the degree of within-cohort trait change were consistent with our predictions that minimal change is favored in harsher climates. At the species level, *Protea punctata* seedlings changed their SLA and photosynthetic rates less than any other studied white protea species. Interestingly, SLA in *P. punctata* also remains more similar between wild seedlings and co-occurring adults than *P. aurea* subsp. *aurea* (Carlson and Holsinger, unpublished), suggesting that this strategy of minimal change is maintained into adulthood. At the population level, we also found that within-cohort change in SLA is smaller (less negative) in populations with colder winter temperature and more intense dry-season drought. Both species and population level findings are consistent with the expectation that trait change should be minimized in the harshest environments [57], [69].

Although *Protea* populations and species differ substantially in the extent to which rainfall is concentrated in winter months, we found no evidence that rainfall seasonality is related to patterns of leaf maturation. This is somewhat surprising given that plasticity is thought to be favored in heterogeneous environments when its costs are low [70]. In our species-level comparison, the steepest rates of change within leaves were observed in *P. aurea* subsp. *aurea*, which is intermediate along the rainfall seasonality axis. The two species that experience the strongest seasonal peaks in rainfall, *P. lacticolor* and *P. mundii*, showed the least change in SLA. At the population-level, we also detected no correlations along the rainfall seasonality axis.

### Conclusions

We find that environmental differences are associated with differences in trait trajectories and their plasticity in white proteas, but differences are more strongly associated with home-site resource availability than with within-year heterogeneity in rainfall. Our earlier work [18] suggested that among-population differences in several of the leaf traits examined here represent adaptive differentiation along environmental axes related to water availability and winter temperatures. Those differences were measured in seedlings (∼6 months old), but they predicted survivorship in experimental gardens. Other work on this group has also shown that some –but not all– species perform or survive best inside their native range, based on reciprocal transplant experiments [17]. Here we demonstrate that species differ in (1) the degree to which traits change both within and between annual leaf cohorts and (2) their degree of plasticity in those trait trajectories. In particular, we show that in some cases, leaf traits change less in plants belonging to species and populations that have harsher home-site conditions. Together, these findings provide compelling evidence that the degree to which traits change over time, in addition to their mean values, have diverged among five *Protea* taxa distributed across strong environmental gradients in the Western Cape. These findings also provide new insights into the relative contribution of plasticity and genetic differentiation in shaping differences among plant populations and species [71].
